# Supplementary material for: Cross-country validation of the Arabic version of the WHO-5 Well-Being Index in non-clinical young adults from six Arab countries
Source: Glob Ment Health (Camb). 2025 Aug 19;12:e95. doi: 10.1017/gmh.2025.10051 (PMC12394027; doi:10.1017/gmh.2025.10051)
Supplement: Fekih-Romdhane et al. supplementary material [file S2054425125100514sup001.docx]

**Table S1. Sociodemographic characteristics of the participants by country.**

|  | **Tunisia (N=617)** | **Lebanon (N=614)** | **Kuwait (N=663)** | **Egypt (N=893)** | **Jordan (N=258)** | **Morocco (N=202)** |
| --- | --- | --- | --- | --- | --- | --- |
| **Age (years)** | 26.68 ± 4.83 | 22.52 ± 4.42 | 22.42 ± 4.15 | 21.39 ± 2.47 | 26.73 ± 5.39 | 23.20 ± 4.76 |
| **Sex** |  |  |  |  |  |  |
| Male | 305 (49.4%) | 210 (34.2%) | 136 (20.5%) | 119 (13.3%) | 103 (39.9%) | 48 (23.8%) |
| Female | 312 (50.6%) | 404 (65.8%) | 527 (79.5%) | 774 (86.7%) | 155 (60.1%) | 154 (76.2%) |
| **Marital status** |  |  |  |  |  |  |
| Married | 240 (38.9%) | 62 (10.1%) | 130 (19.6%) | 101 (11.3%) | 96 (37.2%) | 41 (20.3%) |
| Single | 308 (49.9%) | 540 (87.9%) | 516 (77.8%) | 787 (88.1%) | 155 (60.1%) | 157 (77.7%) |
| Divorced | 60 (9.7%) | 10 (1.6%) | 16 (2.4%) | 5 (0.6%) | 6 (2.3%) | 3 (1.5%) |
| Widowed | 9 (1.5%) | 2 (0.3%) | 1 (0.2%) | 0 (0%) | 1 (0.4%) | 1 (0.5%) |
| **Education** |  |  |  |  |  |  |
| Elementary | 46 (7.5%) | 5 (0.8%) | 0 (0%) | 0 (0%) | 0 (0%) | 0 (0%) |
| Middle | 186 (30.1%) | 5 (0.8%) | 8 (1.2%) | 2 (0.2%) | 6 (2.3%) | 1 (0.5%) |
| High school | 210 (34.0%) | 107 (17.4%) | 60 (9.0%) | 4 (0.4%) | 20 (7.8%) | 6 (3.0%) |
| University | 175 (28.4%) | 497 (80.9%) | 595 (89.7%) | 887 (99.3%) | 232 (89.9%) | 195 (96.5%) |

**Table S2. Standardized Residual Covariances**

|  | **WellBeing5** | **WellBeing4** | **WellBeing3** | **WellBeing2** | **WellBeing1** |
| --- | --- | --- | --- | --- | --- |
| **WellBeing5** | .000 |  |  |  |  |
| **WellBeing4** | -.113 | .000 |  |  |  |
| **WellBeing3** | .270 | .599 | .000 |  |  |
| **WellBeing2** | -.189 | -.599 | -.113 | .000 |  |
| **WellBeing1** | .025 | .000 | -.820 | .952 | .000 |

**Table S3. Modification indices (M.I.)**

|  |  |  | **M.I.** | **Par Change** |
| --- | --- | --- | --- | --- |
| e3 | <--> | e4 | 8.707 | .026 |
| e2 | <--> | e4 | 5.862 | -.022 |
| e1 | <--> | e3 | 19.979 | -.042 |
| e1 | <--> | e2 | 24.417 | .049 |

**Table S4. Country level analyses for concurrent validity.**

**a) Tunisia**

|  | **1** | **2** | **3** | **4** | **5** |
| --- | --- | --- | --- | --- | --- |
| 1. Wellbeing | 1 |  |  |  |  |
| 2. Depression | -.35*** | 1 |  |  |  |
| 3. Anxiety | -.37*** | .92*** | 1 |  |  |
| 4. Stress | -.42*** | .81*** | .83*** | 1 |  |
| 5. Suicidal ideation | -.22*** | -.003 | -.02 | -.11** | 1 |
| 6. Insomnia severity | -.08 | .10* | .09* | .02 | .44*** |

*p < .05; ***p < .001

**b) Lebanon**

|  | **1** | **2** | **3** | **4** | **5** |
| --- | --- | --- | --- | --- | --- |
| 1. Wellbeing | 1 |  |  |  |  |
| 2. Depression | -.26*** | 1 |  |  |  |
| 3. Anxiety | -.27*** | .80*** | 1 |  |  |
| 4. Stress | -.21*** | .78*** | .72*** | 1 |  |
| 5. Suicidal ideation | -.27*** | .28*** | .31*** | .22*** | 1 |
| 6. Insomnia severity | -.49*** | .23*** | .29*** | .22*** | .28*** |

***p < .001

**c) Kuwait**

|  | **1** | **2** | **3** | **4** | **5** |
| --- | --- | --- | --- | --- | --- |
| 1. Wellbeing | 1 |  |  |  |  |
| 2. Depression | -.29*** | 1 |  |  |  |
| 3. Anxiety | -.28*** | .70*** | 1 |  |  |
| 4. Stress | -.25*** | .67*** | .66*** | 1 |  |
| 5. Suicidal ideation | -.21*** | .31*** | .33*** | .17*** | 1 |
| 6. Insomnia severity | -.41*** | .37*** | .41*** | .37*** | .27*** |

***p < .001

**d) Egypt**

|  | **1** | **2** | **3** | **4** | **5** |
| --- | --- | --- | --- | --- | --- |
| 1. Wellbeing | 1 |  |  |  |  |
| 2. Depression | -.24*** | 1 |  |  |  |
| 3. Anxiety | -.22*** | .76*** | 1 |  |  |
| 4. Stress | -.20*** | .78*** | .72*** | 1 |  |
| 5. Suicidal ideation | -.30*** | .20*** | .23*** | .10** | 1 |
| 6. Insomnia severity | -.45*** | .27*** | .30*** | .25*** | .32*** |

**p < .01; ***p < .001

**e) Jordan**

|  | **1** | **2** | **3** | **4** | **5** |
| --- | --- | --- | --- | --- | --- |
| 1. Wellbeing | 1 |  |  |  |  |
| 2. Depression | -.18** | 1 |  |  |  |
| 3. Anxiety | -.17** | .76*** | 1 |  |  |
| 4. Stress | -.14* | .76*** | .72*** | 1 |  |
| 5. Suicidal ideation | -.26*** | .13* | .10 | .07 | 1 |
| 6. Insomnia severity | -.46*** | .11 | .14* | .06 | .27*** |

*p < .05; **p< .01; ***p < .001

**f) Morocco**

|  | **1** | **2** | **3** | **4** | **5** |
| --- | --- | --- | --- | --- | --- |
| 1. Wellbeing | 1 |  |  |  |  |
| 2. Depression | -.51*** | 1 |  |  |  |
| 3. Anxiety | -.45*** | .68*** | 1 |  |  |
| 4. Stress | -.51*** | .61*** | .55*** | 1 |  |
| 5. Suicidal ideation | -.30*** | .37*** | .36*** | .21*** | 1 |
| 6. Insomnia severity | -.51*** | .41*** | .39*** | .50*** | .11 |

***p < .001
